# Supplementary material for: Kinetic modeling predicts a stimulatory role for ribosome collisions at elongation stall sites in bacteria
Source: eLife. 2017 May 12;6:e23629. doi: 10.7554/eLife.23629 (PMC5446239; doi:10.7554/eLife.23629)
Supplement: Supplementary file 1. — DOI: http://dx.doi.org/10.7554/eLife.23629.012 [file elife-23629-supp1.pdf]

Simulation parameters for Fig. 3

**Parameter**	**Value**	**Note**
Stall site identity	CTA	Panel A, B, C
Stall site location (codon number along *yfp*)	46	Panel A
Initiation rate	[0.019, 0.038, 0.075, 0.15, 0.3, 0.6, 1.2, 2.4] s^{-1}	Panel A
Stall site locations (codon number along *yfp*)	15, 46, 68, 141, 201	Panel B, stall sites were added from 5' to 3'.
First stall site location (codon number along *yfp*)	60	Panel C
Second stall site locations (codon number along *yfp*)	63, 64, . . . 238	Panel C
tRNA accommodation rate at stall site (TJ model)	$0.0667s^{-1}$	Panels A, B, C
tRNA accommodation rate at stall site (SAT model)	$0.287s^{-1}$	Panels A, B, C
tRNA accommodation rate at stall site (CSAT model)	$0.0875s^{-1}$	Panels A, B, C

All other parameters have values shown in Supplementary File 6.
