# Supplementary material for: Kinetic modeling predicts a stimulatory role for ribosome collisions at elongation stall sites in bacteria
Source: eLife. 2017 May 12;6:e23629. doi: 10.7554/eLife.23629 (PMC5446239; doi:10.7554/eLife.23629)
Supplement: Supplementary file 2. — DOI: http://dx.doi.org/10.7554/eLife.23629.013 [file elife-23629-supp2.pdf]

Simulation parameters for Fig. 4 (including Figure supplement)

Parameter	Value	Note
Stall site identity	CTA, CTC, CTT, TCG	
Stall site locations (codon number along *yfp*)	46 (6), 68 (10), 141 (14), 201 (18), 147 (5 for Ser TCG)	Different sites were chosen as indicated in title and schematic.
tRNA accommodation rate at CTA68 (TJ model)	$0.07 s^{-1}$	Fit from experiment
tRNA accommodation rate at CTA141 (TJ model)	$0.12 s^{-1}$	Fit from experiment
tRNA accommodation rate at CTA201 (TJ model)	$0.107 s^{-1}$	Fit from experiment
tRNA accommodation rate at CTA46 (TJ model)	$0.091 s^{-1}$	Fit from experiment
tRNA accommodation rate at CTC68 (TJ model)	$0.184 s^{-1}$	Fit from experiment
tRNA accommodation rate at CTC141 (TJ model)	$0.211 s^{-1}$	Fit from experiment
tRNA accommodation rate at CTT68 (TJ model)	$0.0949 s^{-1}$	Fit from experiment
tRNA accommodation rate at CTT46 (TJ model)	$0.133 s^{-1}$	Fit from experiment
tRNA accommodation rate at CTA68 (CSAT model)	$0.0896 s^{-1}$	Fit from experiment
tRNA accommodation rate at CTA141 (CSAT model)	$0.173 s^{-1}$	Fit from experiment
tRNA accommodation rate at CTA201 (CSAT model)	$0.154 s^{-1}$	Fit from experiment
tRNA accommodation rate at CTA46 (CSAT model)	$0.129 s^{-1}$	Fit from experiment
tRNA accommodation rate at CTC68 (CSAT model)	$0.344 s^{-1}$	Fit from experiment
tRNA accommodation rate at CTC141 (CSAT model)	$0.444 s^{-1}$	Fit from experiment
tRNA accommodation rate at CTT68 (CSAT model)	$0.135 s^{-1}$	Fit from experiment
tRNA accommodation rate at CTT46 (CSAT model)	$0.205 s^{-1}$	Fit from experiment
tRNA accommodation rate at CTA68 (SAT model)	$0.292 s^{-1}$	Fit from experiment
tRNA accommodation rate at CTA141 (SAT model)	$0.637 s^{-1}$	Fit from experiment
tRNA accommodation rate at CTA201 (SAT model)	$0.55 s^{-1}$	Fit from experiment
tRNA accommodation rate at CTA46 (SAT model)	$0.444 s^{-1}$	Fit from experiment
tRNA accommodation rate at CTC68 (SAT model)	$1.45 s^{-1}$	Fit from experiment

Continued on next page

Continued from previous page		
**Parameter**	**Value**	**Note**
tRNA accommodation rate at CTC141 (SAT model)	$1.83s^{-1}$	Fit from experiment
tRNA accommodation rate at CTT68 (SAT model)	$0.469s^{-1}$	Fit from experiment
tRNA accommodation rate at CTT46 (SAT model)	$0.713s^{-1}$	Fit from experiment
tRNA accommodation rate at TCG147 (TJ model)	$0.273s^{-1}$	Fit from experiment
tRNA accommodation rate at TCG147 (CSAT model)	$0.767s^{-1}$	Fit from experiment
tRNA accommodation rate at TCG147 (SAT model)	$4.59s^{-1}$	Fit from experiment
Initiation rate of variant 1	$0.101s^{-1}$	Fit from experiment
Initiation rate of variant 2	$0.143s^{-1}$	Fit from experiment
Initiation rate of variant 3	$0.219s^{-1}$	Fit from experiment
Initiation rate of variant 4	$0.3s^{-1}$	Default value
Initiation rate of variant 5	$0.334s^{-1}$	Fit from experiment

All other parameters have values shown in Supplementary File 6.
