# Supplementary material for: Kinetic modeling predicts a stimulatory role for ribosome collisions at elongation stall sites in bacteria
Source: eLife. 2017 May 12;6:e23629. doi: 10.7554/eLife.23629 (PMC5446239; doi:10.7554/eLife.23629)
Supplement: Supplementary file 3. — DOI: http://dx.doi.org/10.7554/eLife.23629.014 [file elife-23629-supp3.pdf]

Simulation parameters for Fig. 5 (including Figure supplement)

**Parameter**	**Value**	**Note**
Stall site identity	CTA, CTC, CTT, TCG	
Stall site locations (codon number along *yfp*)	15 (2), 46 (6), 68 (10), 141 (14), 201 (18)	Different combinations of these sites were chosen as indicated along X-axis.
tRNA accommodation rate at CTA68 (TJ model)	$0.0617 s^{-1}$	Fit from experiment
tRNA accommodation rate at CTA141 (TJ model)	$0.1 s^{-1}$	Fit from experiment
tRNA accommodation rate at CTA201 (TJ model)	$0.0876 s^{-1}$	Fit from experiment
tRNA accommodation rate at CTA15 (TJ model)	$0.0567 s^{-1}$	Fit from experiment
tRNA accommodation rate at CTA46 (TJ model)	$0.0837 s^{-1}$	Fit from experiment
tRNA accommodation rate at CTC68 (TJ model)	$0.267 s^{-1}$	Fit from experiment
tRNA accommodation rate at CTC141 (TJ model)	$0.286 s^{-1}$	Fit from experiment
tRNA accommodation rate at CTC201 (TJ model)	$0.284 s^{-1}$	Fit from experiment
tRNA accommodation rate at CTC15 (TJ model)	$0.137 s^{-1}$	Fit from experiment
tRNA accommodation rate at CTC46 (TJ model)	$0.205 s^{-1}$	Fit from experiment
tRNA accommodation rate at CTT68 (TJ model)	$0.139 s^{-1}$	Fit from experiment
tRNA accommodation rate at CTT141 (TJ model)	$0.27 s^{-1}$	Fit from experiment
tRNA accommodation rate at CTT201 (TJ model)	$0.246 s^{-1}$	Fit from experiment
tRNA accommodation rate at CTT15 (TJ model)	$0.125 s^{-1}$	Fit from experiment
tRNA accommodation rate at CTT46 (TJ model)	$0.172 s^{-1}$	Fit from experiment
tRNA accommodation rate at CTA68 (SAT model)	$0.26 s^{-1}$	Fit from experiment
tRNA accommodation rate at CTA141 (SAT model)	$0.513 s^{-1}$	Fit from experiment
tRNA accommodation rate at CTA201 (SAT model)	$0.404 s^{-1}$	Fit from experiment
tRNA accommodation rate at CTA15 (SAT model)	$0.266 s^{-1}$	Fit from experiment
tRNA accommodation rate at CTA46 (SAT model)	$0.376 s^{-1}$	Fit from experiment
tRNA accommodation rate at CTC68 (SAT model)	$3.06 s^{-1}$	Fit from experiment
		Continued on next page

Continued from previous page

Parameter	Value	Note
tRNA accommodation rate at CTC141 (SAT model)	4.4s^{-1}	Fit from experiment
tRNA accommodation rate at CTC201 (SAT model)	4.6s^{-1}	Fit from experiment
tRNA accommodation rate at CTC15 (SAT model)	0.764s^{-1}	Fit from experiment
tRNA accommodation rate at CTC46 (SAT model)	1.59s^{-1}	Fit from experiment
tRNA accommodation rate at CTT68 (SAT model)	0.777s^{-1}	Fit from experiment
tRNA accommodation rate at CTT141 (SAT model)	3.94s^{-1}	Fit from experiment
tRNA accommodation rate at CTT201 (SAT model)	2.85s^{-1}	Fit from experiment
tRNA accommodation rate at CTT15 (SAT model)	0.649s^{-1}	Fit from experiment
tRNA accommodation rate at CTT46 (SAT model)	1.09s^{-1}	Fit from experiment
tRNA accommodation rate at CTA68 (CSAT model)	0.0769s^{-1}	Fit from experiment
tRNA accommodation rate at CTA141 (CSAT model)	0.147s^{-1}	Fit from experiment
tRNA accommodation rate at CTA201 (CSAT model)	0.123s^{-1}	Fit from experiment
tRNA accommodation rate at CTA15 (CSAT model)	0.0726s^{-1}	Fit from experiment
tRNA accommodation rate at CTA46 (CSAT model)	0.113s^{-1}	Fit from experiment
tRNA accommodation rate at CTC68 (CSAT model)	0.6s^{-1}	Fit from experiment
tRNA accommodation rate at CTC141 (CSAT model)	0.817s^{-1}	Fit from experiment
tRNA accommodation rate at CTC201 (CSAT model)	0.754s^{-1}	Fit from experiment
tRNA accommodation rate at CTC15 (CSAT model)	0.193s^{-1}	Fit from experiment
tRNA accommodation rate at CTC46 (CSAT model)	0.379s^{-1}	Fit from experiment
tRNA accommodation rate at CTT68 (CSAT model)	0.213s^{-1}	Fit from experiment
tRNA accommodation rate at CTT141 (CSAT model)	0.69s^{-1}	Fit from experiment
tRNA accommodation rate at CTT201 (CSAT model)	0.582s^{-1}	Fit from experiment
tRNA accommodation rate at CTT15 (CSAT model)	0.171s^{-1}	Fit from experiment
tRNA accommodation rate at CTT46 (CSAT model)	0.285s^{-1}	Fit from experiment

Continued on next page

Continued from previous page		
**Parameter**	**Value**	**Note**
tRNA accommodation rate at TCG28 (TJ model)	$0.263s^{-1}$	Fit from experiment
tRNA accommodation rate at TCG30 (TJ model)	$0.266s^{-1}$	Fit from experiment
tRNA accommodation rate at TCG86 (TJ model)	$0.281s^{-1}$	Fit from experiment
tRNA accommodation rate at TCG147 (TJ model)	$0.273s^{-1}$	Fit from experiment
tRNA accommodation rate at TCG202 (TJ model)	$0.252s^{-1}$	Fit from experiment
tRNA accommodation rate at TCG205 (TJ model)	$0.29s^{-1}$	Fit from experiment
tRNA accommodation rate at TCG28 (CSAT model)	$0.488s^{-1}$	Fit from experiment
tRNA accommodation rate at TCG30 (CSAT model)	$0.509s^{-1}$	Fit from experiment
tRNA accommodation rate at TCG86 (CSAT model)	$0.725s^{-1}$	Fit from experiment
tRNA accommodation rate at TCG147 (CSAT model)	$0.767s^{-1}$	Fit from experiment
tRNA accommodation rate at TCG202 (CSAT model)	$0.601s^{-1}$	Fit from experiment
tRNA accommodation rate at TCG205 (CSAT model)	$0.935s^{-1}$	Fit from experiment
tRNA accommodation rate at TCG28 (SAT model)	$2.19s^{-1}$	Fit from experiment
tRNA accommodation rate at TCG30 (SAT model)	$2.13s^{-1}$	Fit from experiment
tRNA accommodation rate at TCG86 (SAT model)	$3.82s^{-1}$	Fit from experiment
tRNA accommodation rate at TCG147 (SAT model)	$4.59s^{-1}$	Fit from experiment
tRNA accommodation rate at TCG202 (SAT model)	$3.22s^{-1}$	Fit from experiment
tRNA accommodation rate at TCG205 (SAT model)	$5.15s^{-1}$	Fit from experiment

All other parameters have values shown in Supplementary File 6.
