# Supplementary material for: Kinetic modeling predicts a stimulatory role for ribosome collisions at elongation stall sites in bacteria
Source: eLife. 2017 May 12;6:e23629. doi: 10.7554/eLife.23629 (PMC5446239; doi:10.7554/eLife.23629)
Supplement: Supplementary file 4. — DOI: http://dx.doi.org/10.7554/eLife.23629.015 [file elife-23629-supp4.pdf]

Simulation parameters for Fig. 6 (including Figure supplement)

Parameter	Value	Note
Stall site identity	CTA, CTC	
Stall site locations (codon number along *yfp*)	60 (8),64 (9), 68 (10), 119 (11), 125 (12), 137 (13), 141 (14)	Different combinations of these sites as indicated along X-axis.
tRNA accommodation rate at CTC68 (TJ model)	$0.226 s^{-1}$	Fit from experiment
tRNA accommodation rate at CTC119 (TJ model)	$0.199 s^{-1}$	Fit from experiment
tRNA accommodation rate at CTC125 (TJ model)	$0.205 s^{-1}$	Fit from experiment
tRNA accommodation rate at CTC137 (TJ model)	$0.236 s^{-1}$	Fit from experiment
tRNA accommodation rate at CTC141 (TJ model)	$0.273 s^{-1}$	Fit from experiment
tRNA accommodation rate at CTC60 (TJ model)	$0.228 s^{-1}$	Fit from experiment
tRNA accommodation rate at CTC64 (TJ model)	$0.214 s^{-1}$	Fit from experiment
tRNA accommodation rate at CTC68 (CSAT model)	$0.468 s^{-1}$	Fit from experiment
tRNA accommodation rate at CTC119 (CSAT model)	$0.387 s^{-1}$	Fit from experiment
tRNA accommodation rate at CTC125 (CSAT model)	$0.394 s^{-1}$	Fit from experiment
tRNA accommodation rate at CTC137 (CSAT model)	$0.512 s^{-1}$	Fit from experiment
tRNA accommodation rate at CTC141 (CSAT model)	$0.777 s^{-1}$	Fit from experiment
tRNA accommodation rate at CTC60 (CSAT model)	$0.483 s^{-1}$	Fit from experiment
tRNA accommodation rate at CTC64 (CSAT model)	$0.433 s^{-1}$	Fit from experiment
tRNA accommodation rate at CTC68 (SAT model)	$2.17 s^{-1}$	Fit from experiment
tRNA accommodation rate at CTC119 (SAT model)	$1.71 s^{-1}$	Fit from experiment
tRNA accommodation rate at CTC125 (SAT model)	$1.67 s^{-1}$	Fit from experiment
tRNA accommodation rate at CTC137 (SAT model)	$2.51 s^{-1}$	Fit from experiment
tRNA accommodation rate at CTC141 (SAT model)	$4.26 s^{-1}$	Fit from experiment
tRNA accommodation rate at CTC60 (SAT model)	$2.15 s^{-1}$	Fit from experiment
tRNA accommodation rate at CTC64 (SAT model)	$1.85 s^{-1}$	Fit from experiment

Continued on next page

Continued from previous page		
**Parameter**	**Value**	**Note**
tRNA accommodation rate at CTA68 (CSAT model)	0.0103s^{-1}	Fit from experiment
tRNA accommodation rate at CTA119 (CSAT model)	0.0146s^{-1}	Fit from experiment
tRNA accommodation rate at CTA125 (CSAT model)	0.0158s^{-1}	Fit from experiment
tRNA accommodation rate at CTA137 (CSAT model)	0.0159s^{-1}	Fit from experiment
tRNA accommodation rate at CTA141 (CSAT model)	0.0183s^{-1}	Fit from experiment
tRNA accommodation rate at CTA60 (CSAT model)	0.0183s^{-1}	Fit from experiment
tRNA accommodation rate at CTA64 (CSAT model)	0.012s^{-1}	Fit from experiment
tRNA accommodation rate at CTA68 (TJ model)	0.00825s^{-1}	Fit from experiment
tRNA accommodation rate at CTA119 (TJ model)	0.0112s^{-1}	Fit from experiment
tRNA accommodation rate at CTA125 (TJ model)	0.0113s^{-1}	Fit from experiment
tRNA accommodation rate at CTA137 (TJ model)	0.0116s^{-1}	Fit from experiment
tRNA accommodation rate at CTA141 (TJ model)	0.0127s^{-1}	Fit from experiment
tRNA accommodation rate at CTA60 (TJ model)	0.013s^{-1}	Fit from experiment
tRNA accommodation rate at CTA64 (TJ model)	0.00923s^{-1}	Fit from experiment
tRNA accommodation rate at CTA68 (SAT model)	0.0348s^{-1}	Fit from experiment
tRNA accommodation rate at CTA119 (SAT model)	0.0463s^{-1}	Fit from experiment
tRNA accommodation rate at CTA125 (SAT model)	0.0529s^{-1}	Fit from experiment
tRNA accommodation rate at CTA137 (SAT model)	0.0517s^{-1}	Fit from experiment
tRNA accommodation rate at CTA141 (SAT model)	0.0607s^{-1}	Fit from experiment
tRNA accommodation rate at CTA60 (SAT model)	0.0672s^{-1}	Fit from experiment
tRNA accommodation rate at CTA64 (SAT model)	0.0408s^{-1}	Fit from experiment

All other parameters have values shown in Supplementary File 6.
