# Supplementary material for: Kinetic modeling predicts a stimulatory role for ribosome collisions at elongation stall sites in bacteria
Source: eLife. 2017 May 12;6:e23629. doi: 10.7554/eLife.23629 (PMC5446239; doi:10.7554/eLife.23629)
Supplement: Supplementary file 5. — DOI: http://dx.doi.org/10.7554/eLife.23629.016 [file elife-23629-supp5.pdf]

Simulation parameters for Fig. 7

Parameter	Value	Note
Stall site identity	CTA	Panel A, B
Stall site location (codon number along *yfp*)	201	Panel A, B
Threshold tRNA accommodation rate for selective abortive termination	$22 s^{-1}$	Panel A, B
Threshold tRNA accommodation rate for non-selective abortive termination	$0 s^{-1}$	Panel A, B
tRNA accommodation rate at stall site (TJ model)	$0.0914 s^{-1}$	Panel A, B
tRNA accommodation rate at stall site (SAT model)	$0.425 s^{-1}$	Panel A, B
tRNA accommodation rate at stall site (CSAT model)	$0.13 s^{-1}$	Panel A, B

All other parameters have values shown in Supplementary File 6.
