# Supplementary material for: Kinetic modeling predicts a stimulatory role for ribosome collisions at elongation stall sites in bacteria
Source: eLife. 2017 May 12;6:e23629. doi: 10.7554/eLife.23629 (PMC5446239; doi:10.7554/eLife.23629)
Supplement: Supplementary file 6. — DOI: http://dx.doi.org/10.7554/eLife.23629.017 [file elife-23629-supp6.pdf]

Parameters common to all simulations

Parameter	Value	Units	Reference
Cell volume	10^{-18}	m^{-3}	*Bremer and Dennis* (*1996*); *Elf et al.* (*2003*)
Cell doubling time	30	min	This work
Concentration of tRNAs	4.08×10^5	molecules/cell	*Bremer and Dennis* (*2008*)
Rate of peptide-bond formation and translocation	≈ 22 (1/0.045)	s^{-1}	*Bremer and Dennis* (*2008*)
tRNA accommodation rate at normal sites (not stall sites)	80–1650	s^{-1}	Inferred (Methods)
Ribosome footprint size on mRNAs	10	codons	This work
Number of distinct mRNA species	2	No units	This work
Ribosome-tRNA association rate constant	2×10^7	M^{-1} s^{-1}	*Bilgin and Ehrenberg* (*1994*); *Pavlov and Ehrenberg* (*1996*)
tRNA aminoacylation rate constant	2×10^{10}	M^{-1} s^{-1}	This work
Threshold tRNA accommodation rate for selective abortive termination	22	s^{-1}	This work
Abortive termination rate constant (SAT and CSAT models)	1	s^{-1}	This work
Abortive termination rate constant (TJ model)	0	s^{-1}	This work
Translation initiation rate	0.3	s^{-1}	This work

References

Bilgin N, Ehrenberg M. Mutations in 23 S Ribosomal RNA Perturb Transfer RNA Selection and Can Lead to Streptomycin Dependence. Journal of Molecular Biology. 1994 jan; 235(3):813–824.

Bremer H, Dennis P. Modulation of Chemical Composition and Other Parameters of the Cell at Different Exponential Growth Rates. EcoSal Plus. 2008; doi: 10.1128/ecosal.5.2.3.

Bremer H, Dennis P. Modulation of Chemical Composition and Other Parameters of the Cell by Growth Rate. In: *Escherichia Coli and Salmonella* Washington, DC: ASM Press; 1996.p. 1553–1569.

Elf J, Nilsson D, Tenson T, Ehrenberg M. Selective Charging of tRNA Isoacceptors Explains Patterns of Codon Usage. Science. 2003 jun; 300(5626):1718–1722.

Pavlov MY, Ehrenberg M. Rate of Translation of Natural mRNAs in an Optimized in Vitro System. Archives of Biochemistry and Biophysics. 1996 apr; 328(1):9–16.
