# Supplementary material for: Kinetic modeling predicts a stimulatory role for ribosome collisions at elongation stall sites in bacteria
Source: eLife. 2017 May 12;6:e23629. doi: 10.7554/eLife.23629 (PMC5446239; doi:10.7554/eLife.23629)
Supplement: Supplementary file 7. — DOI: http://dx.doi.org/10.7554/eLife.23629.018 [file elife-23629-supp7.pdf]

List of strains and plasmids

Strain Number	Genotype	Note
ecMF1	MG1655-*attBλ::lacItetRspeR-leuB::FRT*	(**Subramaniam et al., 2013**)
ecMF82	ecMF1 pASEC1 (pZS*11-*yfp0*)	No stall control during Leu starvation, parent *yfp0* sequence, Addgene #53241, **Subramaniam et al.** (**2013**)
ecMF112	ecMF1 pASEC1-CTA4to10	Control with 7 Leu stall sites
ecMF323	ecMF1 pZS*11-3xFLAG-*yfp0*	Fig. 1
ecMF324	ecMF1 pZS*11-3xFLAG-*yfp0*-CTA13	Fig. 1
ecMF325	ecMF1 pZS*11-3xFLAG-*yfp0*-CTA14	Fig. 1
ecMF326	ecMF1 pZS*11-3xFLAG-*yfp0*-CTA13-14	Fig. 1
ecMF327	ecMF1 pZS*11-3xFLAG-*yfp0*-CTA13-14	Fig. 1
ecMF74	ecMF1 pASEC1-CTA18	Fig. 4, 5
ecMF103	ecMF1 pASEC1-TTG	Fig. 4, Fig. 4–Figure supplement 1
ecMF104	ecMF1 pASEC1-ATC	Fig. 4, Fig. 4–Figure supplement 1
ecMF107	ecMF1 pASEC1-RBS2	Fig. 4, Fig. 4–Figure supplement 1
ecMF109	ecMF1 pASEC1-RBS4	Fig. 4, Fig. 4–Figure supplement 1
ecMF152	ecMF1 pASEC1-TTG-CTA18	Fig. 4
ecMF155	ecMF1 pASEC1-ATC-CTA18	Fig. 4
ecMF159	ecMF1 pASEC1-RBS2-CTA18	Fig. 4
ecMF163	ecMF1 pASEC1-RBS4-CTA18	Fig. 4
ecMF62	ecMF1 pASEC1-CTA2	Fig. 5
ecMF66	ecMF1 pASEC1-CTA6	Fig. 5, Fig. 4–Figure supplement 1
ecMF68	ecMF1 pASEC1-CTA10	Fig. 5, Fig. 6, Fig. 4–Figure supplement 1
ecMF72	ecMF1 pASEC1-CTA14	Fig. 5, Fig. 6, Fig. 4–Figure supplement 1
ecMF89	ecMF1 pASEC1-CTA2-6	Fig. 5
ecMF91	ecMF1 pASEC1-CTA2-10	Fig. 5
ecMF83	ecMF1 pASEC1-CTA2-14	Fig. 5
ecMF84	ecMF1 pASEC1-CTA2-18	Fig. 5
ecMF92	ecMF1 pASEC1-CTA6-10	Fig. 5
ecMF85	ecMF1 pASEC1-CTA6-14	Fig. 5
ecMF86	ecMF1 pASEC1-CTA6-18	Fig. 5
ecMF87	ecMF1 pASEC1-CTA10-14	Fig. 5
ecMF88	ecMF1 pASEC1-CTA10-18	Fig. 5
ecMF90	ecMF1 pASEC1-CTA14-18	Fig. 5
ecMF438	ecMF1 pASEC1-CTA6-10-14	Fig. 5
ecMF439	ecMF1 pASEC1-CTA6-10-18	Fig. 5
ecMF440	ecMF1 pASEC1-CTA6-10-14-18	Fig. 5
ecMF80	ecMF1 pASEC1-CTA8	Fig. 6
ecMF67	ecMF1 pASEC1-CTA9	Fig. 6
ecMF69	ecMF1 pASEC1-CTA11	Fig. 6
ecMF70	ecMF1 pASEC1-CTA12	Fig. 6
ecMF71	ecMF1 pASEC1-CTA13	Fig. 6
ecMF249	ecMF1 pASEC1-CTA8-9	Fig. 6
ecMF250	ecMF1 pASEC1-CTA8-10	Fig. 6

Continued on next page

Continued from previous page

Strain Number	Genotype	Note
ecMF251	ecMF1 pASEC1-CTA8-11	Fig. 6
ecMF252	ecMF1 pASEC1-CTA8-12	Fig. 6
ecMF253	ecMF1 pASEC1-CTA8-13	Fig. 6
ecMF254	ecMF1 pASEC1-CTA8-14	Fig. 6
ecMF260	ecMF1 pASEC1-CTA11-12	Fig. 6
ecMF261	ecMF1 pASEC1-CTA13-14	Fig. 6
ecMF99	ecMF1 pASEC1-CTT6	Fig. 4–Figure supplement 1A, Fig. 5–Figure supplement 1B
ecMF135	ecMF1 pASEC1-TTG-CTT6	Fig. 4–Figure supplement 1A
ecMF139	ecMF1 pASEC1-ATC-CTT6	Fig. 4–Figure supplement 1A
ecMF143	ecMF1 pASEC1-RBS2-CTT6	Fig. 4–Figure supplement 1A
ecMF147	ecMF1 pASEC1-RBS4-CTT6	Fig. 4–Figure supplement 1A
ecMF100	ecMF1 pASEC1-CTT10	Fig. 4–Figure supplement 1B, Fig. 5–Figure supplement 1B
ecMF136	ecMF1 pASEC1-TTG-CTT10	Fig. 4–Figure supplement 1B
ecMF140	ecMF1 pASEC1-ATC-CTT10	Fig. 4–Figure supplement 1B
ecMF144	ecMF1 pASEC1-RBS2-CTT10	Fig. 4–Figure supplement 1B
ecMF148	ecMF1 pASEC1-RBS4-CTT10	Fig. 4–Figure supplement 1B
ecMF95	ecMF1 pASEC1-CTC10	Fig. 4–Figure supplement 1C, Fig. 5–Figure supplement 1A, Fig. 6–Figure supplement 1
ecMF133	ecMF1 pASEC1-TTG-CTC10	Fig. 4–Figure supplement 1C
ecMF137	ecMF1 pASEC1-ATC-CTC10	Fig. 4–Figure supplement 1C
ecMF141	ecMF1 pASEC1-RBS2-CTC10	Fig. 4–Figure supplement 1C
ecMF145	ecMF1 pASEC1-RBS4-CTC10	Fig. 4–Figure supplement 1C
ecMF96	ecMF1 pASEC1-CTC14	Fig. 4–Figure supplement 1D, Fig. 5–Figure supplement 1A
ecMF134	ecMF1 pASEC1-TTG-CTC14	Fig. 4–Figure supplement 1D
ecMF138	ecMF1 pASEC1-ATC-CTC14	Fig. 4–Figure supplement 1D
ecMF142	ecMF1 pASEC1-RBS2-CTC14	Fig. 4–Figure supplement 1D
ecMF146	ecMF1 pASEC1-RBS4-CTC14	Fig. 4–Figure supplement 1D
ecMF149	ecMF1 pASEC1-TTG-CTA6	Fig. 4–Figure supplement 1E
ecMF156	ecMF1 pASEC1-RBS2-CTA6	Fig. 4–Figure supplement 1E
ecMF160	ecMF1 pASEC1-RBS4-CTA6	Fig. 4–Figure supplement 1E
ecMF150	ecMF1 pASEC1-TTG-CTA10	Fig. 4–Figure supplement 1F
ecMF153	ecMF1 pASEC1-ATC-CTA10	Fig. 4–Figure supplement 1F
ecMF157	ecMF1 pASEC1-RBS2-CTA10	Fig. 4–Figure supplement 1F
ecMF161	ecMF1 pASEC1-RBS4-CTA10	Fig. 4–Figure supplement 1F
ecMF151	ecMF1 pASEC1-TTG-CTA14	Fig. 4–Figure supplement 1G
ecMF154	ecMF1 pASEC1-ATC-CTA14	Fig. 4–Figure supplement 1G
ecMF158	ecMF1 pASEC1-RBS2-CTA14	Fig. 4–Figure supplement 1G
ecMF162	ecMF1 pASEC1-RBS4-CTA14	Fig. 4–Figure supplement 1G
ecMF403	MG1655-*attBλ::lacItetRspeR-serA::Plac-serA-kanR*	lacI-repressed serA gene
ecMF411	ecMF403 pASEC2 (pZS*11-*yfp13*)	No stall control during Ser starvation, parent *yfp13* sequence, ***Subramaniam et al.*** (***2013***)

Continued on next page

Continued from previous page		
**Strain Number**	**Genotype**	**Note**
ecMF412	ecMF403 pASEC2-TCG2to8	Control with 7 Ser stall sites
ecMF407	ecMF403 pASEC2-TCG5	Fig. 4–Figure supplement 1H, Fig. 5–Figure supplement 1C
ecMF424	ecMF403 pASEC2-TTG-TCG5	Fig. 4–Figure supplement 1H
ecMF428	ecMF403 pASEC2-ATC-TCG5	Fig. 4–Figure supplement 1H
ecMF432	ecMF403 pASEC2-RBS2-TCG5	Fig. 4–Figure supplement 1H
ecMF436	ecMF403 pASEC2-RBS4-TCG5	Fig. 4–Figure supplement 1H
ecMF93	ecMF1 pASEC1-CTC2	Fig. 5–Figure supplement 1A
ecMF94	ecMF1 pASEC1-CTC6	Fig. 5–Figure supplement 1A
ecMF97	ecMF1 pASEC1-CTC18	Fig. 5–Figure supplement 1A
ecMF119	ecMF1 pASEC1-CTC2-6	Fig. 5–Figure supplement 1A
ecMF121	ecMF1 pASEC1-CTC2-10	Fig. 5–Figure supplement 1A
ecMF113	ecMF1 pASEC1-CTC2-14	Fig. 5–Figure supplement 1A
ecMF114	ecMF1 pASEC1-CTC2-18	Fig. 5–Figure supplement 1A
ecMF122	ecMF1 pASEC1-CTC6-10	Fig. 5–Figure supplement 1A
ecMF115	ecMF1 pASEC1-CTC6-14	Fig. 5–Figure supplement 1A
ecMF116	ecMF1 pASEC1-CTC6-18	Fig. 5–Figure supplement 1A
ecMF117	ecMF1 pASEC1-CTC10-14	Fig. 5–Figure supplement 1A
ecMF118	ecMF1 pASEC1-CTC10-18	Fig. 5–Figure supplement 1A
ecMF120	ecMF1 pASEC1-CTC14-18	Fig. 5–Figure supplement 1A
ecMF98	ecMF1 pASEC1-CTT2	Fig. 5–Figure supplement 1B
ecMF101	ecMF1 pASEC1-CTT14	Fig. 5–Figure supplement 1B
ecMF102	ecMF1 pASEC1-CTT18	Fig. 5–Figure supplement 1B
ecMF129	ecMF1 pASEC1-CTT2-6	Fig. 5–Figure supplement 1B
ecMF131	ecMF1 pASEC1-CTT2-10	Fig. 5–Figure supplement 1B
ecMF123	ecMF1 pASEC1-CTT2-14	Fig. 5–Figure supplement 1B
ecMF124	ecMF1 pASEC1-CTT2-18	Fig. 5–Figure supplement 1B
ecMF132	ecMF1 pASEC1-CTT6-10	Fig. 5–Figure supplement 1B
ecMF125	ecMF1 pASEC1-CTT6-14	Fig. 5–Figure supplement 1B
ecMF126	ecMF1 pASEC1-CTT6-18	Fig. 5–Figure supplement 1B
ecMF127	ecMF1 pASEC1-CTT10-14	Fig. 5–Figure supplement 1B
ecMF128	ecMF1 pASEC1-CTT10-18	Fig. 5–Figure supplement 1B
ecMF130	ecMF1 pASEC1-CTT14-18	Fig. 5–Figure supplement 1B
ecMF404	ecMF403 pASEC2-TCG2	Fig. 5–Figure supplement 1C
ecMF405	ecMF403 pASEC2-TCG3	Fig. 5–Figure supplement 1C
ecMF406	ecMF403 pASEC2-TCG4	Fig. 5–Figure supplement 1C
ecMF408	ecMF403 pASEC2-TCG6	Fig. 5–Figure supplement 1C
ecMF409	ecMF403 pASEC2-TCG7	Fig. 5–Figure supplement 1C
ecMF413	ecMF403 pASEC2-TCG2-5	Fig. 5–Figure supplement 1C
ecMF414	ecMF403 pASEC2-TCG2-6	Fig. 5–Figure supplement 1C
ecMF415	ecMF403 pASEC2-TCG2-7	Fig. 5–Figure supplement 1C
ecMF416	ecMF403 pASEC2-TCG3-5	Fig. 5–Figure supplement 1C
ecMF417	ecMF403 pASEC2-TCG3-6	Fig. 5–Figure supplement 1C
ecMF418	ecMF403 pASEC2-TCG3-7	Fig. 5–Figure supplement 1C
ecMF419	ecMF403 pASEC2-TCG4-5	Fig. 5–Figure supplement 1C
ecMF420	ecMF403 pASEC2-TCG4-6	Fig. 5–Figure supplement 1C
ecMF421	ecMF403 pASEC2-TCG4-7	Fig. 5–Figure supplement 1C
		Continued on next page

Continued from previous page

Strain Number	Genotype	Note
ecMF164	ecMF1 pASEC1-CTC8	Fig. 6–Figure supplement 1
ecMF165	ecMF1 pASEC1-CTC9	Fig. 6–Figure supplement 1
ecMF226	ecMF1 pASEC1-CTC11	Fig. 6–Figure supplement 1
ecMF227	ecMF1 pASEC1-CTC12	Fig. 6–Figure supplement 1
ecMF166	ecMF1 pASEC1-CTC8-9	Fig. 6–Figure supplement 1
ecMF167	ecMF1 pASEC1-CTC8-10	Fig. 6–Figure supplement 1
ecMF228	ecMF1 pASEC1-CTC8-11	Fig. 6–Figure supplement 1
ecMF229	ecMF1 pASEC1-CTC8-12	Fig. 6–Figure supplement 1
ecMF168	ecMF1 pASEC1-CTC9-10	Fig. 6–Figure supplement 1
ecMF234	ecMF1 pASEC1-CTC9-11	Fig. 6–Figure supplement 1
ecMF235	ecMF1 pASEC1-CTC9-12	Fig. 6–Figure supplement 1
ecMF230	ecMF1 pASEC1-CTC11-12	Fig. 6–Figure supplement 1
ecMF395	MG1655-*leuB::FRT-attBλ::yfp0-ampR*	Author-response Fig. 1, 2
ecMF396	MG1655-*leuB::FRT-attBλ::yfp0-CTA4to10-ampR*	Author-response Fig. 1, 2
ecMF397	MG1655-*leuB::FRT-attBλ::yfp0-CTA6-ampR*	Author-response Fig. 1, 2
ecMF398	MG1655-*leuB::FRT-attBλ::yfp0-CTA10-ampR*	Author-response Fig. 1, 2
ecMF399	MG1655-*leuB::FRT-attBλ::yfp0-CTA14-ampR*	Author-response Fig. 1, 2
ecMF400	MG1655-*leuB::FRT-attBλ::yfp0-CTA6-10-ampR*	Author-response Fig. 1, 2
ecMF401	MG1655-*leuB::FRT-attBλ::yfp0-CTA6-14-ampR*	Author-response Fig. 1, 2
ecMF402	MG1655-*leuB::FRT-attBλ::yfp0-CTA10-14-ampR*	Author-response Fig. 1, 2

References

Subramaniam AR, Pan T, Cluzel P. Environmental Perturbations Lift the Degeneracy of the Genetic Code to Regulate Protein Levels in Bacteria. PNAS. 2013; 110(6):2419–2424.
